# Evaluation of adjuvant therapy for T1-2N1miM0 breast cancer without further axillary lymph node dissection

**DOI:** 10.3389/fsurg.2022.905437

**Published:** 2023-01-06

**Authors:** Baiyu Li, Jianbo Liu, Guangyin Wu, Qingyao Zhu, Shundong Cang

**Affiliations:** ^1^ Department of Oncology, Henan Provincial People's Hospital, Zhengzhou, China; ^2^ Department of Oncology, People's Hospital of Zhengzhou University, Zhengzhou, China; ^3^ Department of Oncology, People's Hospital of Henan, University, Zhengzhou, China

**Keywords:** sentinel lymph nodes micrometastases, adjuvant chemotherapy, adjuvant radiotherapy, breast cancer, sentinel lymph biopsy

## Abstract

**Background:**

For breast cancer (BC) with sentinel lymph node micrometastases (SLNMs), there are limited data to guide the selection of postoperative adjuvant therapy. This study aimed to identify target populations who might benefit most from adjuvant therapy and examine prognostic factors among patients with T1-2N1miM0 BC with one or two SLNMs who underwent sentinel lymph node biopsy (SLNB) alone.

**Methods:**

There were 7,423 patients diagnosed with T1-2N1miM0 BC between 2010 and 2015, and patients with one or two SLNMs were extracted from the Surveillance, Epidemiology, and End Results database. All the patients underwent SLNB alone without further axillary lymph node dissection, and they were stratified according to adjuvant therapy. The statistical significance of categorical variables was analyzed using the *χ*^2^ test. Univariable and multivariable Cox analyses were used to analyze characteristics predictive of Breast-cancer-specific survival and overall survival (OS). Kaplan–Meier methods with the log-rank test was analyzed to compare survival difference between the different treatments.

**Results:**

Adjuvant chemotherapy and radiotherapy improved 5-year OS rates. Multivariate analysis revealed that age ≥70 years, high grade, T2 stage, triple-negative subtype, and absence of radiotherapy were poor prognostic factors for OS. Patients who received breast-conserving surgery (BCS), and those with invasive ductal carcinoma (IDC), luminal A, luminal B, or basal-like subtype, and T1c or T2 stage benefited from adjuvant radiotherapy. Patients who received BCS, and those with IDC, luminal A subtype, and T1b, T1c, or T2 stage benefited from adjuvant chemotherapy.

**Conclusion:**

Our findings provide a clinical evaluation of treatment choice after surgery, which may help clinicians make individualized clinical decisions.

## Introduction

A growing number of sentinel lymph node micrometastases (SLNMs) have been detected by the widespread application of SLN biopsy (SLNB) and advances in immunohistochemical detection for pathological diagnosis (1). For patients with micrometastases, previous studies, such as the AATRM (2) and IBCSG23-01 (3) trials, have supported de-escalation of axillary surgery, that is, SLNB without further axillary lymph node dissection (ALND). Compared with ALND, SLNB can accurately reflect (4, 5) ALN status and significantly reduce complications following ALND, such as lymphedema (6). Patients undergoing SLNB need postoperative adjuvant therapy. However, the prognosis and indications for postoperative adjuvant therapy in patients with SLNMs are still controversial. The IBCSG 23-01 (3) and ACOSOG Z0011 (7) trials recommended that postoperative adjuvant therapy including radiotherapy, chemotherapy, and endocrine therapy should be strengthened, but the above studies still had some limitations. The IBCSG 23-01 (3) trial showed better disease-free survival in patients who accepted any postoperative systemic therapy compared with those without such therapy. Ninety-one percent of patients received breast-conserving surgery (BCS) and whole-breast radiation in the IBCSG 23-01 trial. The number of patients receiving mastectomies was small. Accordingly, larger study populations may be required to identify whether patients receiving mastectomy can benefit from adjuvant therapy.

The ACOSOG Z0011 (7) trial showed that 10-year overall survival (OS) in patients with early breast cancer (BC) and one or two SLNMs who underwent SLNB and postoperative adjuvant therapy was not inferior to OS in those who underwent ALND. The trial highlighted the need for postoperative adjuvant therapy, but HER2 status was not clear and there was insufficient evidence that the above findings could be applied similarly to all different molecular types of BC.

To help fill this lack of evidence, we investigated the role of adjuvant therapy in women with different molecular types of BC with one or two SLNMs who underwent SLNB alone. We also analyzed clinicopathological parameters, such as pathological grade, race, and T stage, which are known to contribute individually to prognosis. We searched the Surveillance, Epidemiology, and End Results (SEER) database for the indications for chemotherapy or radiotherapy and found high-risk factors that affected prognosis.

## Materials and methods

### Data acquisition and patient selection

There were 7,423 patients diagnosed with BC based on the International Classification of Disease for Oncology. The data for this study were acquired from the April 2019 release of the SEER database and were analyzed using SEER*stat version 8.3.6 software. We selected patients who were pathologically diagnosed with BC between 2010 and 2015 from 18 SEER registries, representing 33% of the USA population. Women with pT1-2 N1micM0 BC [American Joint Committee on Cancer staging system, 7th edition (34)] were included in the present study. The following histological codes from the third revision of the International Classification of Diseases were included: 8,500/3 [invasive ductal carcinoma (IDC)], 8,520/3 [invasive lobular carcinoma (ILC)], 8,522/3 [mixed invasive ductal and lobular carcinoma (IDLC)], and 8,523/3 (IDC mixed with other types of carcinoma).

The exclusion criteria were as follows: male patients; >5 regional nodes examined; >2 positive nodes obtained; patients with secondary tumors or incomplete data; and patients who did not accept mastectomy or BCS.

The following variables were retrieved for analysis: age; marital status; race; tumor grade, laterality, and histological type; surgery (mastectomy or BCS); adjuvant chemotherapy (yes or no); tumor molecular subtype and T stage; radiotherapy (yes or no); and molecular typing (estrogen receptor (ER), progesterone receptor (PR) and HER2 hormone receptor status). Grade III BC included poorly differentiated and undifferentiated histological grades. The SEER database did not categorize the axillary procedures performed. Therefore, surrogates were used to classify patients as having undergone SLNB or ALND. We defined SLNB if 1–5 LNs were removed and as ALND if >5 LNs were excised (8). The molecular subtypes were differentiated into (9): luminal A (ER and/or PR positive, HER2 negative); luminal B (ER and/or PR positive, HER2 positive), HER2 (HER2 positive, ER, and PR negative); and triple-negative (ER, PR, and HER2 all negative).

### Statistical analysis

Breast cancer-specific survival (BCSS) and OS were the primary endpoints. OS referred to the survival time from diagnosis to death from any cause, and BCSS referred to the survival time from diagnosis to death related to the tumor. The *χ*^2^ test was used to test categorical variables. Univariate and multivariate Cox regression was carried out for OS and BCSS to identify independent risk factors. Variables with *P* < 0.1 in the univariate model were included in the multivariate model. Kaplan–Meier methods with the log-rank test was analyzed to compare survival difference between the different treatments. Subgroup analyses were also carried out to determine whether adjuvant therapy could benefit different groups.

*P* < 0.05 was defined as statistically significant. All statistical analyses were calculated using SPSS version 20.0 (SPSS Inc., Chicago, IL, United States). The R programming language (version 3.3.1) was used to make forest plots. We used GraphPad Prism (version 7.0) software to generate Kaplan–Meier survival graphs.

## Results

### Baseline characteristics

We analyzed 7,423 women with a diagnosis of pT1-2N1mic BC with one or two SLNMs between 2010 and 2015. We divided the treatments into the following four categories: no adjuvant therapy, adjuvant chemotherapy, adjuvant radiotherapy, and both adjuvant therapies (Table 1). Adjuvant treatments (radiotherapy, chemotherapy, or both) were performed more frequently in patients younger (<35) (respectively 6.7%, 26.1%, 50.4%), aged >70 years (respectively 44.9%, 6.8%, 9.9%), with a grade III–IV BC (14.5%, 29.8%, 38.9%) and underwent to BCS (47.8%, 9.6%, 31.0%), while, underwent to mastectomy (7.3%, 32.3%, 14.5%). pT1a (35.1%) and luminal A (26.9%) BC had a major percentage of no adjuvant therapy. Unexpectedly, adjuvant therapy was performed more often in cases with two rather than one SLNM.

**Table 1:** Patient and tumor characteristics, stratified by adjuvant treatments.

Characteristics	Therapy				*P-*value
	No adjuvant therapy	Adjuvant chemotherapy	Both adjuvant therapies	Adjuvant radiotherapy	
*N*	1,836	1,357	1,830	2,400	
Age					0.000
<35	20 (16.8%)	31 (26.1%)	60 (50.4%)	8 (6.7%)	
35–50	314 (18.8%)	483 (28.9%)	572 (34.2%)	302 (18.1%)	
50–70	878 (21.9%)	733 (18.3%)	1,037 (25.9%)	1,362 (34.0%)	
>70	624 (38.4%)	110 (6.8%)	161 (9.90%)	728 (44.9%)	
Marital status					0.000
Married	970 (21.9%)	849 (19.2%)	1,178 (26.6%)	1,432 (32.3%)	
Single	238 (24.3%)	199 (20.3%)	258 (26.4%)	284 (29.0%)	
Divorced, separated, or windowed	513 (30.6%)	242 (14.4%)	317 (18.9%)	606 (36.1%)	
Unknown	115 (34.1%)	67 (19.9%)	77 (22.8%)	78 (3.3%)	
Race					0.000
White	1,528 (25.1%)	1,073 (17.6%)	1,456 (23.9%)	2,023 (33.3%)	
Black	142 (21.7%)	134 (20.5%)	198 (30.2%)	181 (27.6%)	
Others	166 (24.1%)	150 (21.8%)	176 (25.6%)	196 (28.5%)	
Tumor grade					0.000
Grade I	489 (29.0%)	156 (9.30%)	244 (14.5%)	797 (47.3%)	
Grade II	1,023 (26.9%)	625 (16.4%)	834 (21.9%)	1,323 (34.8%)	
Grade III/IV	324 (16.8%)	576 (29.8%)	752 (38.9%)	280 (14.5%)	
Laterality					0.627
Left	953 (25.4%)	681 (18.1%)	921 (24.5%)	1,200 (32.0%)	
Right	883 (24.1%)	676 (18.4%)	909 (24.8%)	1,200 (32.7%)	
Histological type					0.000
IDC	1,407 (23.6%)	1,118 (18.8%)	1,543 (25.9%)	1,891 (31.7%)	
ILC	197 (29.1%)	98 (14.5%)	125 (18.5%)	256 (37.9%)	
IDLC	164 (29.9%)	95 (17.3%)	117 (21.4%)	172 (31.4%)	
IDC with other types of carcinoma	68 (28.3%)	46 (19.2%)	45 (18.8%)	81 (33.8%)	
Surgery					0.000
BCS	531 (11.6%)	441 (9.6%)	1,420 (31.0%)	2,194 (47.8%)	
Mastectomy	1,305 (46.0%)	916 (32.3%)	410 (14.5%)	206 (7.3%)	
Subtype					0.000
Luminal A	1,661 (26.9%)	910 (14.7%)	1,293 (20.9%)	2,322 (37.5%)	
Luminal B	94 (14.8%)	220 (34.6%)	269 (42.4%)	52 (8.2%)	
HER2	24 (14.0%)	85 (49.7%)	60 (35.1%)	2 (1.2%)	
Basal-like	57 (13.2%)	142 (32.9%)	208 (48.3%)	24 (5.6%)	
pT					0.000
T1a	84 (35.1%)	45 (18.8%)	34 (14.2%)	76 (31.8%)	
T1b	291 (27.4%)	137 (12.9%)	190 (17.9%)	445 (41.9%)	
T1c	810 (24.1%)	540 (16.0%)	741 (22.0%)	1,275 (37.9%)	
T2	651 (23.6%)	635 (23.0%)	865 (31.4%)	604 (21.9%)	
pN1mi					0.000
One micrometastasis	1,732 (25.3%)	1,234 (18.0%)	1,650 (24.1%)	2,235 (32.6%)	
Two micrometastasis	104 (18.2%)	123 (21.5%)	180 (31.5%)	165 (28.8%)	

IDC, invasive ductal carcinoma; ILC, invasive lobular carcinoma; IDLC, mixed invasive ductal and lobular carcinoma; BCS, breast-conserving surgery.

### Univariate and multivariate analysis of overall survival

Variables with *P* < 0.1 in the univariate analysis included age, marital status, tumor grade, histology, surgery, tumor subtype, T stage, and chemotherapy or radiotherapy (Table 2). All these variables were included in the multivariate model. After adjustment for confounding factors, multivariate analysis indicated that age, marital status, race, behavior, tumor grade, tumor subtype, T stage, and radiotherapy were maintained. In the final multivariate Cox regression, poorer OS was associated with: age ≥70 years (adjusted hazard ratios (AHR) 2.728; 95% CI, 1.095–6.792; *P* = 0.031); poorly differentiated and undifferentiated histology (AHR, 1.711; 95% CI, 1.215–2.408; *P* = 0.002); stage T2 (AHR, 3.188; 95% CI, 1.175–8.650; *P* = 0.023); triple-negative subtype (AHR, 1.997; 95% CI, 1.417–2.816; *P* = 0.000); single marital status (AHR, 1.676; 95% CI, 1.217–2.307; *P* = 0.002); or divorced (AHR, 1.703; 95% CI, 1.327–2.184; *P* = 0.000). IDLC (AHR, 0.570; 95% CI, 0.337–0.962; *P* = 0.035) predicted better OS. Nonreceipt of radiotherapy was correlated with decreased OS (AHR, 1.846; 95% CI, 1.423–2.394; *P* = 0.000), but nonreceipt of chemotherapy was not associated with decreased OS (AHR, 1.200; 95% CI, 0.921–1.563; *P* = 0.176).

**Table 2:** Univariate and multivariate Cox analysis of overall survival in patients who had one or two sentinel lymph node micrometastases and underwent sentinel lymph node biopsy alone.

Characteristics	Univariate analysis	Variables with a *P-*value < 0.1 in the univariable model were included in the multivariable model	Multivariable analysis	
	OS HR (95% CI)	*P-*value	OS AHR (95% CI)	*P-*value
Age				
<35	1		1	
35–50	0.457 (0.178–1.174)	0.104	0.535 (0.207–1.382)	0.197
50–70	0.802 (0.329–1.958)	0.628	0.957 (0.388–2.360)	0.924
>70	2.642 (1.085–6.429)	0.032	2.728 (1.095–6.792)	0.031
Marital status				
Married	1		1	
Single	1.723 (1.255–2.365)	0.001	1.676 (1.217–2.307)	0.002
Divorced, separated, or windowed	2.660 (2.098–3.372)	0.000	1.703 (1.327–2.184)	0.000
Unknown	1.811 (1.121–2.926)	0.015	1.351 (0.832–2.194)	0.224
Race				
White	1		/	/
Black	1.229 (0.873–1.731)	0.238	/	/
Others	0.728 (0.472–1.124)	0.152	/	/
Tumor grade				
Grade I	1		1	
Grade II	1.089 (0.805–1.472)	0.581	1.008 (0.743–1.368)	0.958
Grade III/IV	1.989 (1.466–2.700)	0.000	1.711 (1.215–2.408)	0.002
Laterality				
Left	1		/	
Right	0.901 (0.729–1.113)	0.333	/	/
Histological type				
IDC	1		1	
ILC	1.100 (0.774–1.562)	0.596	1.123 (0.781–1.615)	0.531
IDLC	0.554 (0.330–0.931)	0.026	0.570 (0.337–0.962)	0.035
IDC with other types of carcinoma	0.906 (0.482–1.702)	0.759	0.701 (0.372–1.321)	0.271
Surgery				
BCS	1		1	
Mastectomy	1.540 (1.247–1.902)	0.000	1.066 (0.828–1.373)	0.619
Chemotherapy				
Yes	1		1	
No/unknown	1.471 (1.181–1.832)	0.001	1.200 (0.921–1.563)	0.176
Subtype				
Luminal A	1		1	
Luminal B	0.967 (0.642–1.458)	0.873	0.935 (0.610–1.431)	0.755
HER-2	0.772 (0.344–1.734)	0.530	.582 (0.253–1.339)	0.203
Basal-like	2.836 (2.101–3.828)	0.000	1.997 (1.417–2.816)	0.000
pT				
T1a	1		1	
T1b	1.666 (0.587–4.730)	0.337	1.470 (0.517–4.184)	0.470
T1c	2.462 (0.911–6.656)	0.076	2.184 (0.805–5.923)	0.125
T2	4.057 (1.506–10.932)	0.006	3.188 (1.175–8.650)	0.023
pN1mi				
One micrometastasis	1			
Two micrometastasis	0.856 (0.565–1.295)	0.461	/	/
Radiotherapy				
No	2.101 (1.691–2.611)	0.000	1.846 (1.423–2.394)	0.000
Yes	1		1	

SLN, sentinel lymph nodal; SLNB, sentinel lymph node biopsy; IDC, invasive ductal carcinoma; ILC, invasive lobular carcinoma; IDLC, mixed invasive ductal and lobular carcinoma; BCS, breast-conserving surgery; OS, overall survival; SLNMs, sentinel lymph node micrometastases.

### Univariate and multivariate analysis of breast cancer-specific survival

Variables with *P* < 0.1 in our univariate analysis included age, marital status, race, tumor grade, histology, surgery, tumor subtype, T stage, and receipt of chemotherapy (Table 3). All of these variables were included in the multivariate model. After adjustment for confounding factors, multivariate analysis indicated that tumor grade and subtype were maintained. Compared with patients with the luminal A subtype, those with the triple-negative subtype (AHR, 2.654; 95% CI, 1.617–4.358; *P* = 0.000) had poorer BCSS. Compared with patients with well-differentiated histology, patients with poorly differentiated and undifferentiated histology had decreased BCSS (AHR 6.163, *P* = 0.000). Conversely, Luminal B subtype predicted better BCSS (AHR, 0.263; 95% CI, 0.082–0.848; *P* = 0.025). Multivariate analyses also revealed that BCSS was not affected by: the type of surgery (AHR, 1.425; 95% CI, 0.968–2.099; *P* = 0.073); chemotherapy (AHR, 0.890; 95% CI, 0.556–1.424; *P* = 0.628), T stage (all *P* > 0.05); pathological type (all *P* > 0.05); and age (all *P* > 0.05).

**Table 3:** Univariate and multivariate Cox analysis of breast-cancer-specific survival in patients who had one or two sentinel lymph node micrometastases and underwent sentinel lymph node biopsy alone.

Characteristics	Univariate analysis	Variables with a *P-*value < 0.1 in the univariable model were included in the multivariable model	Multivariable analysis	
	BCSS HR (95% CI)	*P-*value	BCSS AHR (95% CI)	*P-*value
Age				
<35	1		1	
35–50	0.286 (0.107–0.762)	0.012	0.415 (0.153–1.125)	0.084
50–70	0.297 (0.119–0.744)	0.010	0.481 (0.187–1.236)	0.129
>70	0.470 (0.182–1.212)	0.118	0.916 (0.333–2.515)	0.864
Marital status				
Married	1		1	
Single	1.582 (0.949–2.636)	0.078	1.344 (0.793–2.279)	0.272
Divorced, separated, or windowed	1.342 (0.852–2.112)	0.204	1.012 (0.626–1.637)	0.961
Unknown	0.465 (0.114–1.907)	0.288	0.333 (0.081–1.369)	0.127
Race				
White	1		1	
Black	1.511 (0.860–2.656)	0.151	1.033 (0.572–1.867)	0.914
Others	0.434 (0.159–1.182)	0.103	0.404 (0.148–1.106)	0.078
Tumor grade				
Grade I	1		1	
Grade II	2.305 (0.964–5.513)	0.061	2.067 (0.860–4.971)	0.105
Grade III/IV	9.752 (4.232–22.472)	0.000	6.163 (2.551–14.889)	0.000
Laterality				
Left	1		/	/
Right	1.071 (0.732–1.568)	0.723	/	/
Histological type				
IDC	1		1	
ILC	0.653 (0.303–1.406)	0.276	0.842 (0.381–1.864)	0.672
IDLC	0.109 (0.015–0.782)	0.028	0.148 (0.020–1.065)	0.058
IDC with other types of carcinoma	0.538 (0.133–2.183)	0.386	0.500 (0.123–2.037)	0.333
Surgery				
BCS	1		1	
Mastectomy	1.544 (1.055–2.260)	0.025	1.425 (0.968–2.099)	0.073
Chemotherapy				
Yes	1		1	
No/unknown	0.562 (0.381–0.830)	0.004	0.890 (0.556–1.424)	0.628
Subtype				
Luminal A	1		1	
Luminal B	0.439 (0.138–1.394)	0.162	0.263 (0.082–0.848)	0.025
HER-2	1.431 (0.450–4.544)	0.544	0.592 (0.180–1.942)	0.387
Basal-like	6.284 (4.097–9.640)	0.000	2.654 (1.617–4.358)	0.000
pT				
T1a	1		1	
T1b	1.337 (0.161–11.102)	0.788	1.225 (0.147–10.246)	0.851
T1c	2.542 (0.348–18.553)	0.358	2.021 (0.275–14.862)	0.490
T2	5.981 (0.830–43.117)	0.076	3.673 (0.503–26.808)	0.199
pN1mi				
One micrometastasis	1		/	/
Two micrometastasis	1.589 (0.889–2.838)	0.118	/	/
Radiotherapy				
No	1.345 (0.919–1.968)	0.128	/	/
Yes	1		/	/

SLN, sentinel lymph nodal; IDC, invasive ductal carcinoma; ILC, invasive lobular carcinoma; IDLC, mixed invasive ductal and lobular carcinoma; BCS, breast-conserving surgery. BCSS, breast-cancer-specific survival; SLNB, sentinel lymph node biopsy; SLNMs, sentinel lymph node micrometastases.

Univariate analyses revealed that radiotherapy had no significant effect on BCSS (AHR, 1.345; 95% CI, 0.919–1.968; *P* = 0.128) (Table 3).

### Subgroup analysis

**Figure 1:**
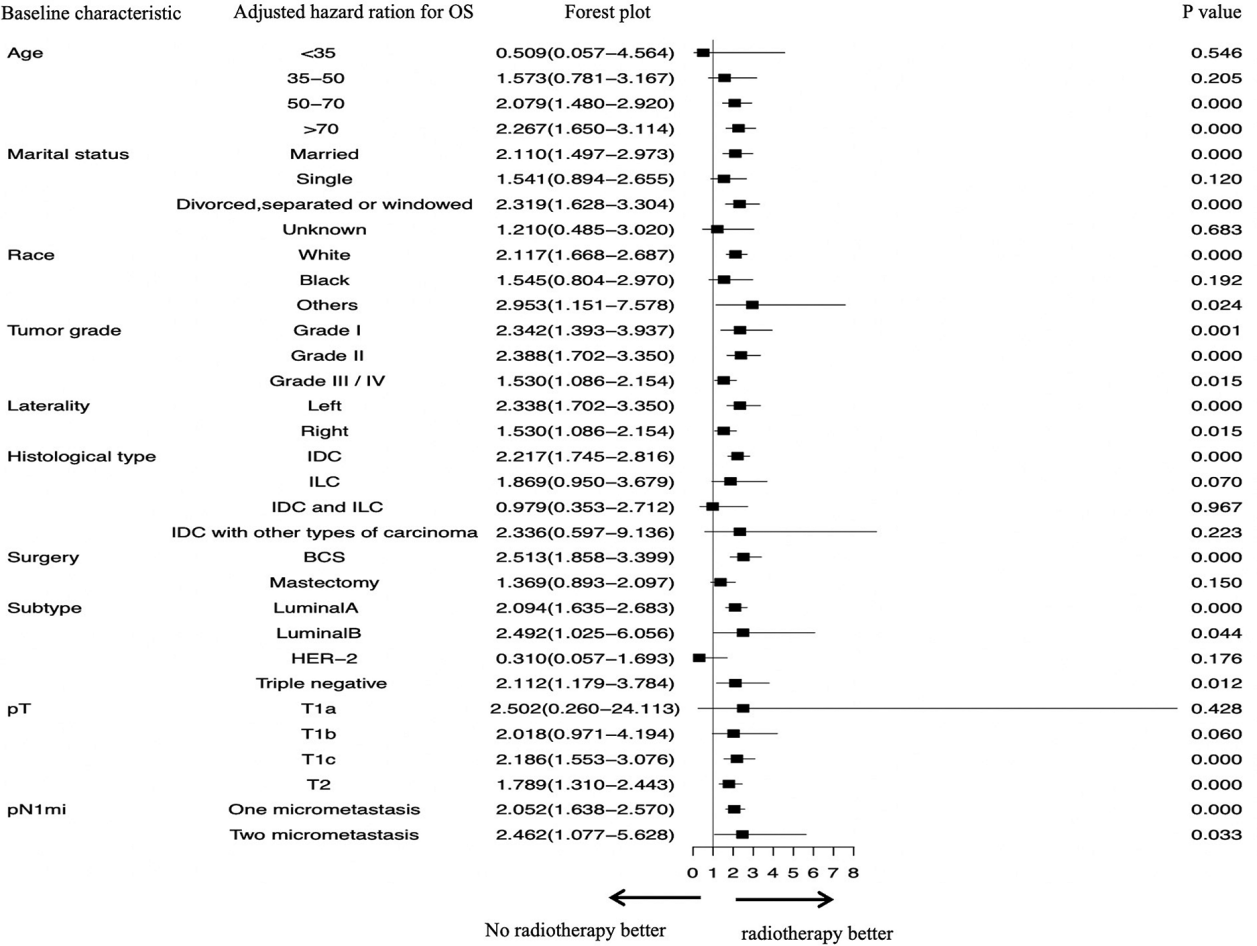
Subgroup analyses assessing the benefit of radiotherapy in patients who had one or two positive sentinel lymph node micrometastases and underwent sentinel lymph node biopsy alone. SLN, sentinel lymph nodal; SLNB, sentinel lymph node biopsy; IDC, invasive ductal carcinoma; ILC, invasive lobular carcinoma; IDLC, mixed invasive ductal and lobular carcinoma; BCS, breast-conserving surgery.

**Figure 2:**
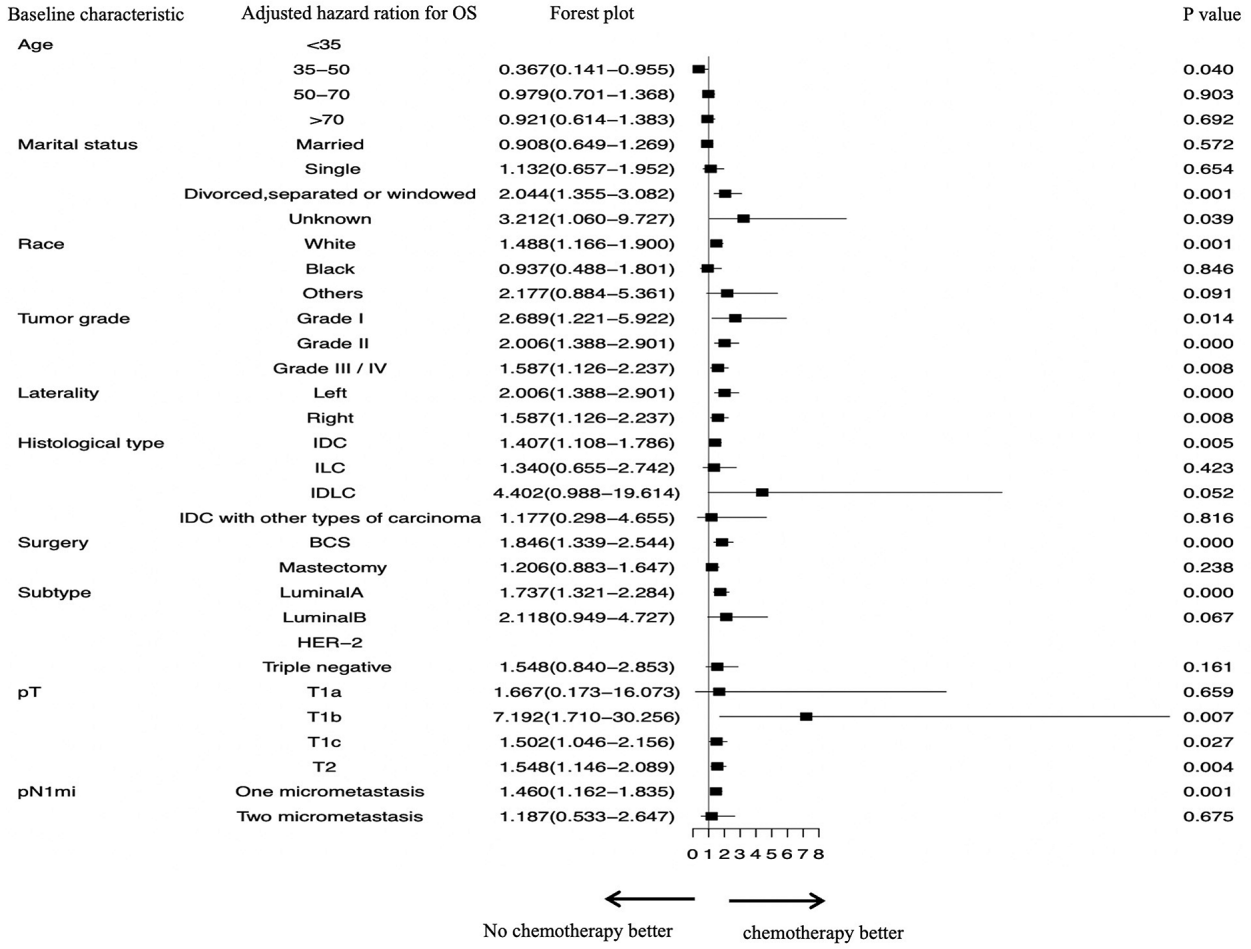
Subgroup analyses assessing the benefit of chemotherapy in patients who had one or two positive sentinel lymph node micrometastases and underwent sentinel lymph node biopsy alone. SLN, sentinel lymph nodal; SLNB, sentinel lymph node biopsy; IDC, invasive ductal carcinoma; ILC, invasive lobular carcinoma; IDLC, mixed invasive ductal and lobular carcinoma; BCS, breast-conserving surgery.

To explore the specific subgroups that may benefit from chemotherapy or radiotherapy after surgery, subgroup analyses were carried out in the matched cohort (Figures 1, 2).

We identified which specific subgroups may benefit from radiotherapy (Figure 1). We found that OS was significantly better in patients who did not accept radiotherapy and in those aged <35 years (*P* = 0.546). However, OS was significantly improved in patients who accepted radiotherapy and in those aged >50 years (50–70 years, AHR, 2.079; *P* = 0.000; >70 years, AHR, 2.267; *P* = 0.000). The following factors contributed to significantly increased OS in patients who received radiotherapy: IDC (AHR, 2.217; *P* = 0.000); grade I–IV (all *P* < 0.05); tumor located left or right (both *P* < 0.05); undergoing BCS (AHR, 2.513; *P* = 0.000); luminal A subtype (AHR, 2.094; *P* = 0.000); luminal B subtype (AHR, 2.492; *P* = 0.044); basal-like subtype (AHR, 2.112; *P* = 0.012); T1c stage (AHR, 2.186; *P* = 0.000); T2 stage (AHR, 1.789; *P* = 0.000); and one or two SLNMs (both *P* < 0.05).

As for chemotherapy, stratified survival analysis showed that OS of nonreceipt of chemotherapy was elevated in patients aged 35–50 years who did not receive chemotherapy (*P* = 0.040) (Figure 2). The following factors were also associated with better OS in patients who received chemotherapy: IDC (AHR, 1.407; *P* = 0.005); grade I–IV (all *P* < 0.05); tumor located left or right (both *P* < 0.05); undergoing BCS (AHR, 1.846; *P* = 0.000); luminal A subtype (AHR, 1.737; *P* = 0.000); T1b stage (AHR, 7.192; *P* = 0.007); T1c stage (AHR, 1.502; *P* = 0.027); T2 stage (AHR, 1.548; *P* = 0.004); and one positive SLNM (AHR, 1.460; *P* = 0.001).

**Figure 3:**
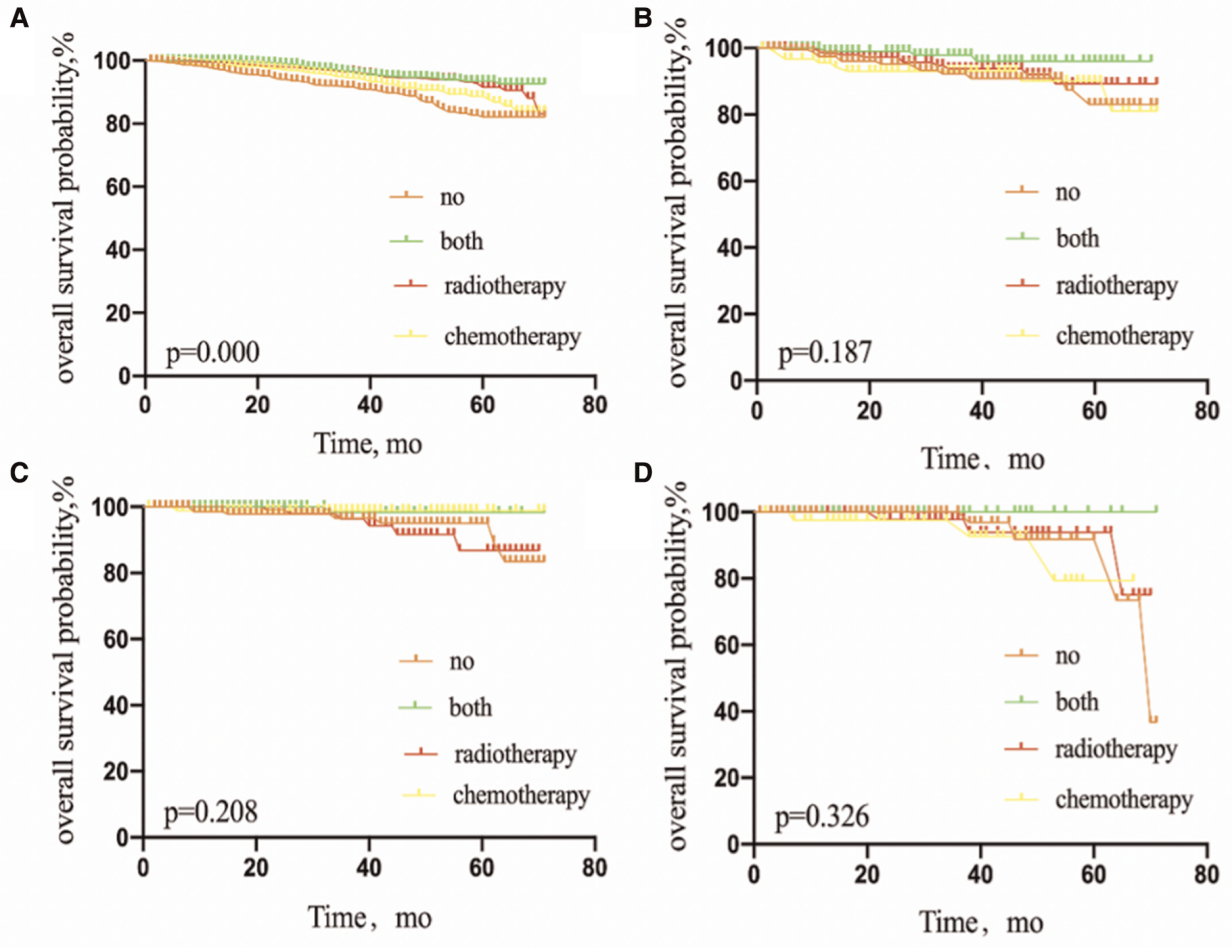
Kaplan–Meier curves depicting overall survival between treatment arms in patients with IDC (**A**), invasive lobular carcinoma (**B**), invasive ductal and lobular carcinoma (**C**), or IDC mixed with other types of carcinoma (**D**) with 1 or 2 sentinel lymph node micrometastases treated with sentinel lymph node biopsy alone. IDC, invasive ductal carcinoma; NO patients treated with no adjuvant therapies; BOTH patients treated with both adjuvant chemotherapy and radiotherapy; RADIOTHERAPY patients treated with adjuvant radiotherapy; CHEMOTHERAPY patients treated with adjuvant chemotherapy.

We performed a Kaplan–Meier analysis to further examine prognostic factors. Patients with IDC benefited from adjuvant therapy (*P* = 0.000), compared with other pathological types, such as ILC (*P* = 0.187), IDLC (*P* = 0.208), and IDC mixed with other types of carcinoma (*P* = 0.326) (Figure 3).

**Figure 4:**
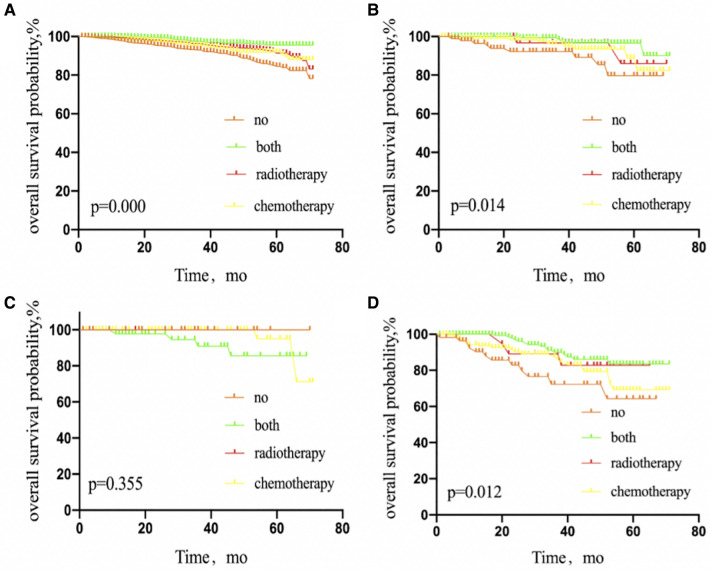
Kaplan–Meier curves depicting overall survival between treatment arms in patients with breast cancer luminal A subtype (**A**), luminal B subtype (**B**), HER2 overexpression subtype (**C**), or triple-negative subtype (**D**) with 1 or 2 sentinel lymph node micrometastases treated with sentinel lymph node biopsy alone. NO patients treated with no adjuvant therapies; BOTH patients treated with both adjuvant chemotherapy and radiotherapy; RADIOTHERAPY patients treated with adjuvant radiotherapy; CHEMOTHERAPY patients treated with adjuvant chemotherapy.

Interestingly, luminal A, luminal B, and triple-negative BC patients benefited from adjuvant therapy (*P* = 0.000, 0.014, and 0.012, respectively), but not patients with HER2 subtype (*P* = 0.355) (Figure 4).

**Figure 5:**
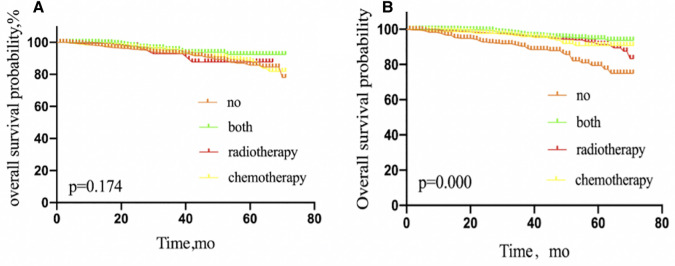
Kaplan–Meier curves depicting overall survival between treatment arms stratified by mastectomy (**A**) or breast-conserving surgery (**B**) in patients with breast cancer with sentinel lymph node micrometastases treated with sentinel lymph node biopsy alone. NO patients treated with no adjuvant therapies; BOTH patients treated with both adjuvant chemotherapy and radiotherapy; RADIOTHERAPY patients treated with adjuvant radiotherapy; CHEMOTHERAPY patients treated with adjuvant chemotherapy.

Compared with patients receiving mastectomy (*P* = 0.174), the survival rate of patients treated with BCS (*P* = 0.000) was increased by adjuvant therapy (Figure 5).

**Figure 6:**
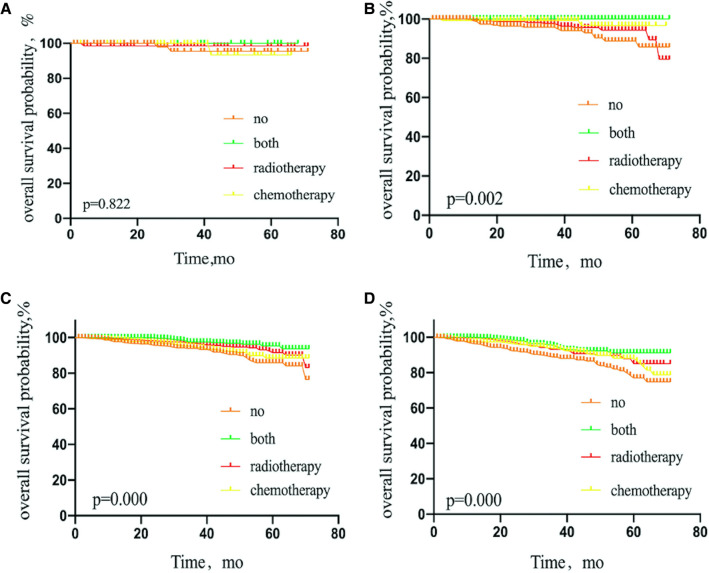
Kaplan–Meier curves depicting overall survival between treatment arms in patients with T1a stage (**A**), T1b stage (**B**), T1c stage (**C**), or T2 stage (**D**) breast cancer with sentinel lymph node micrometastases. NO patients treated with no adjuvant therapies; BOTH patients treated with both adjuvant chemotherapy and radiotherapy; RADIOTHERAPY patients treated with adjuvant radiotherapy; CHEMOTHERAPY patients treated with adjuvant chemotherapy.

Patients with T1a stage tumors who were treated with adjuvant radiotherapy, chemotherapy, or both showed no significant difference in OS, compared with patients without adjuvant therapy (*P* = 0.822). However, patients with T1b, T1c, or T2 stage tumors treated with adjuvant radiotherapy, chemotherapy, or both did show a significantly elevated OS, compared with the untreated group (*P* = 0.002, 0.000, and 0.000, respectively) (Figure 6).

### Survival analysis

**Figure 7:**
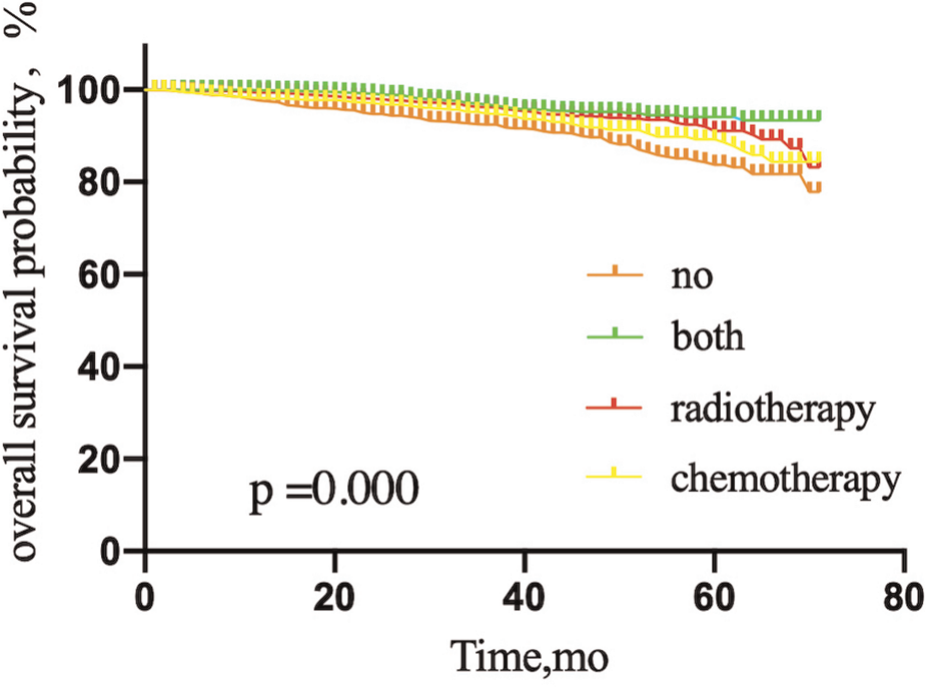
Overall survival probability of patients with micrometastases in 1 or 2 sentinel lymph node micrometastases treated with sentinel lymph node biopsy alone, according to adjuvant therapy. NO patients treated with no adjuvant therapies; BOTH patients treated with both adjuvant chemotherapy and radiotherapy; RADIOTHERAPY patients treated with adjuvant radiotherapy; CHEMOTHERAPY patients treated with adjuvant chemotherapy.

**Figure 8:**
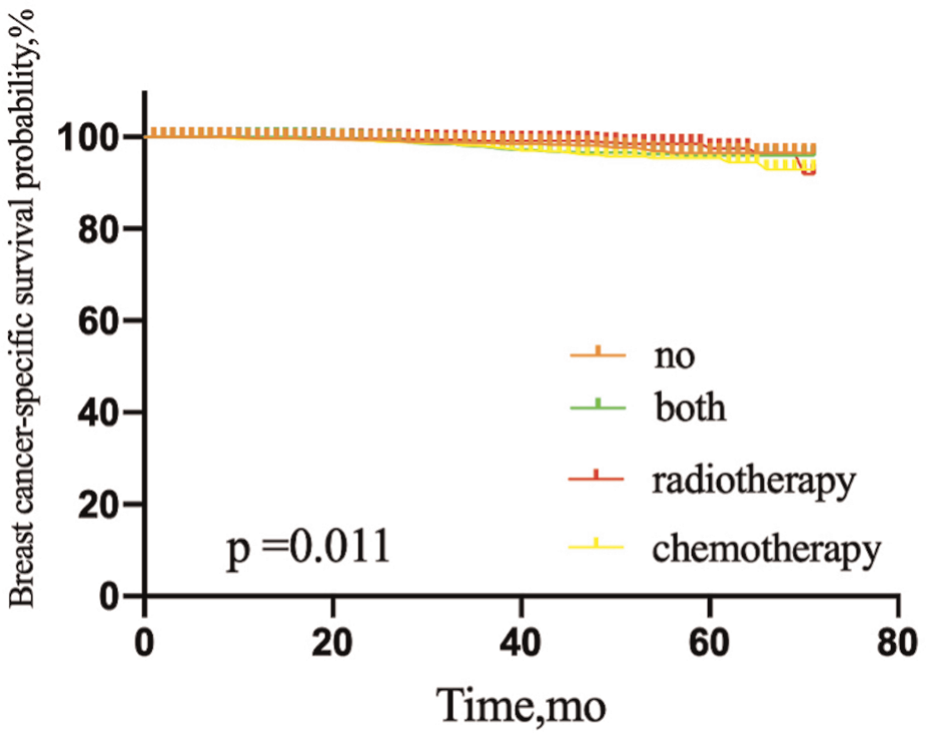
Breast-cancer-specific survival of patients with 1 or 2 sentinel lymph node micrometastases treated with sentinel lymph node biopsy alone, according to adjuvant therapy. NO patients treated with no adjuvant therapies; BOTH patients treated with both adjuvant chemotherapy and radiotherapy; RADIOTHERAPY patients treated with adjuvant radiotherapy; CHEMOTHERAPY patients treated with adjuvant chemotherapy.

The 5-year OS rate among patients with one or two SLNMs was 83.7% in those treated with SLNB alone without adjuvant therapy, 89.3% in the adjuvant chemotherapy group, 94.1% in those treated with both adjuvant therapies, and 91.1% in the adjuvant radiotherapy group (*P* = 0.000) (Figure 7). For BCSS, the corresponding 5-year OS rates were 96.5%, 95.4%, 95.9%, and 97.5%, respectively (*P* = 0.011) (Figure 8).

## Discussion

In the era of precision medicine, the treatment of BC is in transition toward a precise, minimally invasive, and individualized approach (6). The current challenge is how to establish individualized treatment protocols for patients with SLNMs, which was the aim of the present study.

Our multivariate analysis revealed that age ≥70 years, high grade, T2 stage, triple-negative subtype, and no adjuvant radiotherapy were poor prognostic factors for OS. Marital status was also a predictor of OS, single or divorced marital status predicted poor survival, which agrees with the study of Lan, who found that divorced or widowed marital status showed the highest mortality risk among BC patients with IDC (33).

Pepels and Lupe reported (10, 11) poor prognostic factors include tumor size, young age, hormone receptor with negative status, no adjuvant radiotherapy, lymphovascular invasion, and histological grade III.

These high-risk factors need to be paid attention to. Our study found that old age predicted poor prognosis, while previous studies suggested that young age predicted poor prognosis, the different findings about age need further exploration.

The current surgical consensus (12) states that patients with early BC with one or two SLNMs should complete appropriate adjuvant radiotherapy without further ALND. Our study was in line with this consensus. We demonstrated that patients with SLNB plus adjuvant radiotherapy, with or without chemotherapy, had better OS than patients in the adjuvant chemotherapy and untreated groups. Simultaneously, the OS of patients in the adjuvant chemotherapy group was better than that of patients in the untreated group. This result may be interpreted as follows. In the Z0011 (7) and IBCSG23-01 (3) trials, the incidence of non-SLN metastasis in patients with SLNMs was 10% and 13%, respectively. Non-SLN (3) metastasis is one of the possible causes of axillary relapse in patients with SLNMs. Yet, the 10-year follow-up in the IBCSG 23-01 trial (3) showed that the ALN recurrence rate was 2% in patients with SLNMs at stage T1-2 without ALND. Additionally, in the study of Reed et al. (13), patients with SLNMs had a higher rate of distant metastasis after surgery than those without SLNMs. It was speculated that patients with SLNMs not undergoing ALND had a higher rate of axillary non-SLNs and distant metastases, which might have affected OS. The authors concluded that, in these patients, ALND and systemic therapy should be considered. We observed distinct heterogeneity in the treatment between the IBCSG 23-01and AATRM (2) trials. This heterogeneity raises many questions about which patients would benefit most from adjuvant therapy among those with T1-2N1miM0 BC treated with SLNB alone.

Patients were stratified according to surgical procedures to identify target individuals who might benefit most from postoperative adjuvant therapy. The primary surgical procedures for BC include BCS and mastectomy, and the surgical method is associated with prognosis (18). In our study, patients undergoing radiotherapy after BCS had a significantly better prognosis than those who performed a mastectomy. Previous studies (3, 7) indicated that, although 27% of patients had non-SLN involvement, for those who underwent SLNB alone, the regional recurrence rate was only 0.9%. An important explanation for the above phenomenon was that tangential field, whole-breast irradiation contributed to partial coverage of lower ALNs, thereby clearing the residual tumor cells in the ALNs (14). In contrast, our subgroup analysis suggested that patients do not benefit from postmastectomy radiotherapy (PMRT). This is consistent with previous retrospective studies in which PMRT did not improve the local recurrence rate of patients with SLNMs (15, 17). Nevertheless, there was an increasing survival trend in the PMRT group (*P* = 0.053) with only a follow-up of 23.3 months in the study by Wu et al. (16). Similarly, Sun et al. (18) considered that completion of PMRT was correlated with improved OS. The Swedish SENOMIC study (19) showed that patients with SLNMs who underwent mastectomy had a significantly higher recurrence rate than those who received BCS. Merfeld's study (20) proposed that PMRT should not be routinely recommended for patients with micrometastatic disease, but should be recommended for patients with grade III histology. Additional prospective and extended follow-up studies are required to identify whether PMRT is beneficial, and who will benefit most.

We sought to establish whether age affected the selection of adjuvant therapy. We found a significant improvement in OS when patients aged >50 years received radiotherapy. It is generally believed that diagnosis of BC before 50 years of age is a high-risk prognostic factor (21); therefore, radiotherapy might be considered in those patients. The reason for the conclusion of our study may be related to the short survival time among patients who were older than 50 years.

Patients with different molecular subtyping had different biological characteristics. Some researchers (21) believe that the triple-negative subtype is not a high-burden tumor, and is related to distant metastasis, but rarely to regional recurrence. In contrast, the HER-2 subtype has a higher tumor burden than the triple-negative subtype and is more prone to metastasize to ≥4 LNs. The indications for adjuvant chemoradiotherapy also differ. Generally, tumors with a high metastatic rate are more likely to benefit from chemotherapy, and those with a low local recurrence rate are less likely to benefit from radiotherapy. Kaplan–Meier survival curve analysis in our study revealed that patients with the HER-2 subtype did not achieve a benefit from adjuvant radiotherapy or chemotherapy, yet those with triple-negative BC did benefit from adjuvant therapy. HER-2-positive BC may benefit from targeted therapy, which needs confirmation in a prospective, larger study.

St Gallen 2013 Consensus Guidelines Panel announced that the validity of chemotherapy did not rely on the number of positive nodes, but rather on the underlying tumor biology. We concluded that one rather than two positive nodes benefited from chemotherapy. The relationship between chemotherapy and the number of SLNs needs to be further explored.

Conversely, the effectiveness of radiotherapy did not depend on the number of positive SLNMs. Houvenaeghel et al. (22) believed that LNMs might not be necessary when deciding on adjuvant chemotherapy for ER-positive BC. This is in agreement with Hetterich (23). Accordingly, further investigation is needed to explore the relationship between the number of positive SLNMs in patients with different molecular subtyping and adjuvant chemoradiotherapy.

Prior studies have illustrated the relationship between different pathological types of BC and outcomes. Yang et al. (24) reported that ILC and IDC patients had similar OS. Zhao et al. (25) found that ILC and IDC demonstrated better BCSS and OS than IDLC according to multivariate analysis. Our results were consistent with those of Yang, but IDLC exhibited better OS than IDC did. Our study also showed that IDC benefited more than other pathological types from adjuvant therapy. It was shown previously (26) that IDC patients had a significantly higher rate of ALN metastasis compared with patients with ILC or other histological types, which may explain this phenomenon.

Guiding treatment selection among patients with BC and SLNMs according to pathological T stage has been rarely studied. We showed that pT1c and pT2 stages were beneficial for adjuvant radiotherapy, while the pT1b, pT1c, and pT2 stages were beneficial for adjuvant chemotherapy. The pT1a stage did not benefit from both therapies. Our results require further verification. Moreover, the relationship between T staging and radiotherapy based on surgical methods or LN status (27) needs further study. The effect of Ki67 and 21-gene recurrence score (28) on chemotherapy selection also should be discussed in the future.

The present study had several limitations. First, the study was restricted by its retrospective nature, and it was vulnerable to selection bias, in contrast to randomized controlled clinical trials. Second, its clinical and therapeutic features were limited to the USA. Third, details regarding radiotherapy such as dose, treatment volume, and radiation field were not available. Fourth, BC with SLNMs follows indolent behavior and persists for a long time (29, 30). Consequently, longer follow-up is needed (31). Finally, the SEER (32) data did not record treatments such as immunotherapy, targeted therapy, and endocrine therapy, which might have an association with prognosis.

However, this study also had the following strengths. Although the incidence of SLNMs was low, we had a high number of patients who met the enrollment criteria compared with other studies, and we predicted which breast patients may benefit from adjuvant therapy.

## Conclusion

Systematic and comprehensive individualized treatment regimens are needed after surgery in patients with BC and SLNMs. Tumor size, molecular subtyping, histopathological type, and type of surgery may affect the choice of adjuvant therapy. Our findings provide a basis for stratifying patients for radiotherapy and chemotherapy, to direct more personalized and refined therapy decisions. In the future, we need a more prospective, larger sample study with longer follow-ups to validate our results.

## Data Availability

The original contributions presented in the study are included in the article/Supplementary Material, further inquiries can be directed to the corresponding author/s.
